# Anti-Obesity Effects of Hispidin and *Alpinia zerumbet* Bioactives in 3T3-L1 Adipocytes

**DOI:** 10.3390/molecules191016656

**Published:** 2014-10-15

**Authors:** Pham Thi Be Tu, Shinkichi Tawata

**Affiliations:** 1 Department of Bioscience and Biotechnology, The United Graduate School of Agricultural Sciences, Kagoshima University, Korimoto 1-21-24, Kagoshima 890-8580, Japan; E-Mail: ptbetu@yahoo.com; 2 Department of Bioscience and Biotechnology, Faculty of Agriculture, University of the Ryukyus, Senbaru 1, Nishihara-cho, Okinawa 903-0129, Japan

**Keywords:** *Alpinia zerumbet*, 5,6-dehydrokawain, dihydro-5,6-dehydrokawain, hispidin, anti-obesity, 3T3-L1 adipocytes

## Abstract

Obesity and its related disorders have become leading metabolic diseases. In the present study, we used 3T3-L1 adipocytes to investigate the anti-obesity activity of hispidin and two related compounds that were isolated from *Alpinia zerumbet* (alpinia) rhizomes. The results showed that hispidin, dihydro-5,6-dehydrokawain (DDK), and 5,6-dehydrokawain (DK) have promising anti-obesity properties. In particular, all three compounds significantly increased intracellular cyclic adenosine monophosphate (cAMP) concentrations by 81.2% ± 0.06%, 67.0% ± 1.62%, and 56.9% ± 0.19%, respectively. Hispidin also stimulated glycerol release by 276.4% ± 0.8% and inhibited lipid accumulation by 47.8% ± 0.16%. Hispidin and DDK decreased intracellular triglyceride content by 79.5% ± 1.37% and 70.2% ± 1.4%, respectively, and all three compounds inhibited glycerol-3-phosphate dehydrogenase (GPDH) and pancreatic lipase, with hispidin and DDK being the most potent inhibitors. Finally, none of the three compounds reduced 3T3-L1 adipocyte viability. These results highlight the potential for developing hispidin and its derivatives as anti-obesity compounds.

## 1. Introduction

Currently, obesity is the leading metabolic disease throughout the world [1] and is closely associated with coronary heart disease, hypertension, type 2 diabetes mellitus, cancer, respiratory complications, and osteoarthritis [2]. The World Health Organization reports that at least one billion adults are overweight of which 300 million are obese, and these numbers are expected to rise without intervention [3]. The obesity epidemic now affects children too, and the prevalence of childhood obesity has tripled in the past 30 years, leading to health problems in this susceptible population [4]. Moreover, evidence from epidemiological and life-insurance actuarial data indicates that obesity is a strong predictor of decreased longevity [5]. Thus, prevention and treatment of obesity are of critical urgency and importance for improving our quality of life [6]. Recent reports have proposed mechanisms to reduce obesity, including increased lipolysis and inhibited dietary fat absorption. Therefore, stimulating lipolysis and inhibiting fat accumulation studies are increasingly interest for the treatment of obesity.

**Figure 1:**
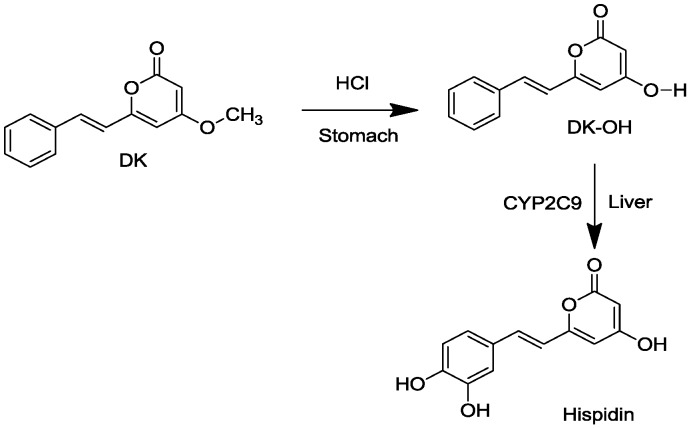
Conversion of 5,6-dehydrokawain (DK) to hispidin.

Hispidin, 6-(3,4-dihydroxylstyryl)-4-hydroxy-2-pyrone, is a phenolic compound derived from the medicinal mushroom *Phellinus linteus* that has protective activity against peroxynitrite-mediated DNA damage and hydroxyl radical generation [7] and protects pancreatic β-cells from hydrogen peroxide damage [8]. Hispidin also has strong antioxidant, anticancer, anti-neuraminidase properties and attenuates carbon tetrachloride-induced hepatotoxicity [9,10,11,12]. Our laboratory has reported that hispidin can be derived from 5,6-dehydrokawain (DK) hydrolysis in the stomach and subsequent metabolism in rabbit liver microsomes that contain CYP2C9 (Figure 1) [13,14]. DK is a known antifungal and integrase inhibitor.

*Alpinia zerumbet* (Pers.) Burtt and Smith (alpinia) is a species within the Zingiberaceae ginger family and is widely distributed in subtropical and tropical regions around of the world [15]. The alpinia plant is a traditional herbal medicine used for its anti-hypertensive, antihyperlocomotion, antipsychotic, antioxidant, vasorelaxant, antidiabetic, and psychopharmacological properties [16,17,18,19,20]. Alpinia has been shown to contain DK, dihydro-5,6-dehydrokawain (DDK), and labdadiene. Alpinia extracts inhibit HIV integrase, neuraminidase, and multiple enzymes related skinning disease [21,22]. DK and DDK also have antiulcerogenic and antithrombotic activity and inhibit aggregation and ATP release from rabbit platelets [23,24]. Given the diverse activities of its bioactive components, there is considerable interest in the further evaluation of the medicinal properties of alpinia.

In the present study, we used 3T3-L1 adipocytes to evaluate whether hispidin and two bioactive components isolated from alpinia rhizomes have potential anti-obesity activity. We first analyzed whether the compounds alter the intracellular concentration of cyclic adenosine monophosphate (cAMP) or stimulate the glycerol release. We then assessed the effects of the compounds on the suppression of lipid accumulation, reduction of triglyceride content, and inhibition of glycerol-3-phosphate dehydrogenase (GPDH) and pancreatic lipase. Finally, the cytotoxicity of the compounds toward 3T3-L1 cells was evaluated.

## 2. Results and Discussion

### 2.1. Conversion of DK to Hispidin and Purification of DK and DDK

**Figure 2:**
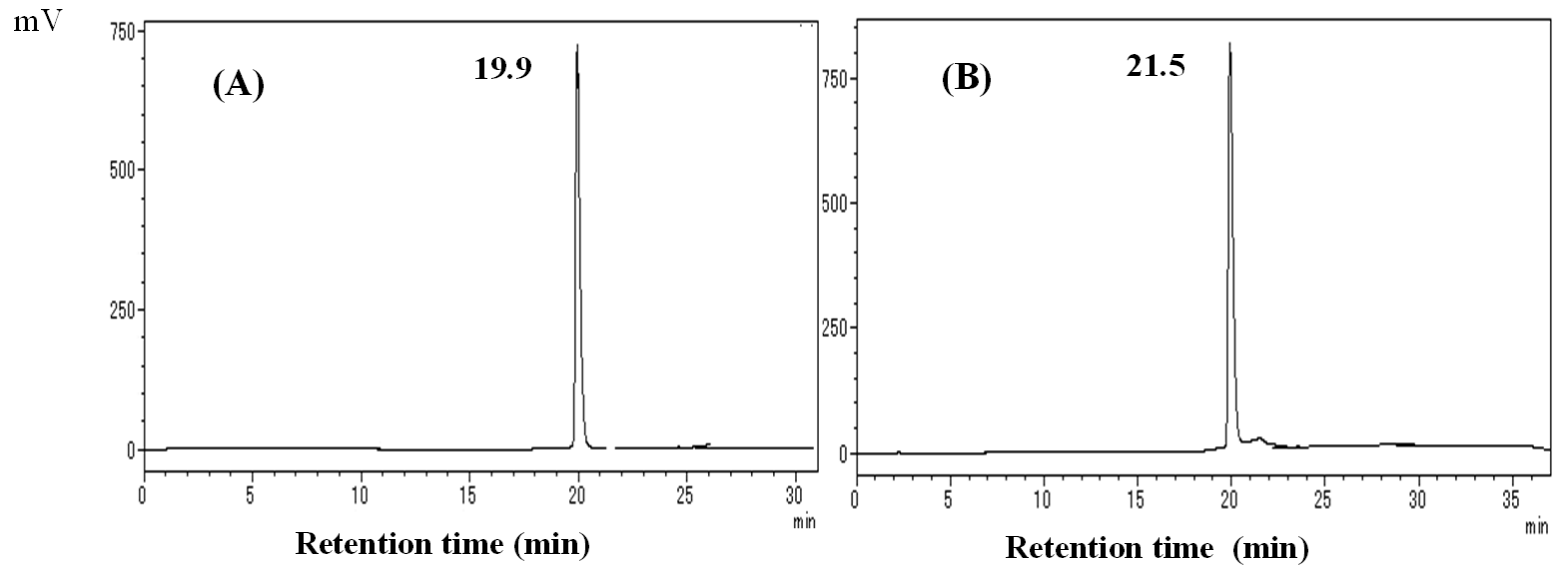
The analytical high performance liquid chromatography (HPLC) chromatograms of *A. zerumbet* rhizomes (**A**) DK, (**B**) dihydro-5,6-dehydrokawain (DDK). HPLC column: ODS-100Z column (5 µm, 150 × 46 mm i.d.). Flow rate: 0.8 mL/min. Injection volume: 5 µL. Monitored wavelength: 280 nm.

Hispidin was produced by hydrolysis of DK in a stomach acid and subsequent metabolism by CYP2C9 in rabbit liver microsomes (Figure 1). Purified DK was eluted with a retention time of 19.9 min in HPLC analysis and DDK with a retention time of 21.5 min (Figure 2).

### 2.2. Effect of Hispidin, DK and DDK on Cell Viability

**Figure 3:**
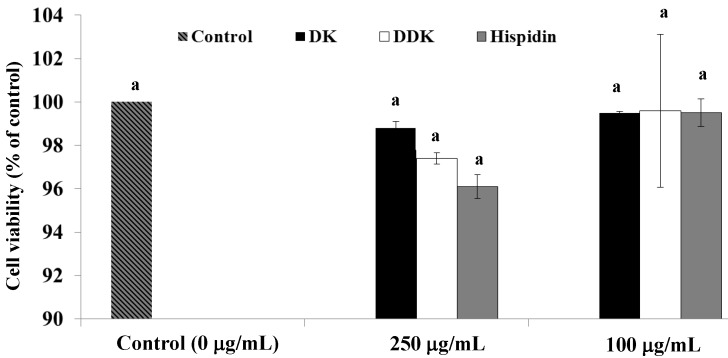
The effect of hispidin, DK and DDK on cell viability in 3T3-L1 adipocytes. Differentiated 3T3-L1 cells were treated with various concentrations of isolated compounds and incubated for 72 h at 37 °C in a humidified incubator containing 5% CO_2_. Data are expressed as the percent growth rate of cells cultured in the presence of samples compared with untreated control cells (mean ± SE; *n* = 3). Letters with different superscripts indicate samples that are significantly different (*p* < 0.05) than the control.

To examine the effect of hispidin, DK and DDK on cell viability, differentiated 3T3-L1 adipocytes were treated with 100 and 250 µg/mL of each compound and incubated at 37 °C with 5% CO_2_ for 72 h. Viability was determined by MTT assay. None of the tested compounds showed a significant effect on 3T3-L1 viability (Figure 3). At 250 µg/mL hispidin, DDK and DK only reduced cell viability by 3.83% ± 0.31%, 2.58% ± 0.26%, and 1.11% ± 0.56%, respectively, compared to the control.

### 2.3. Stimulation of Glycerol Release

**Figure 4:**
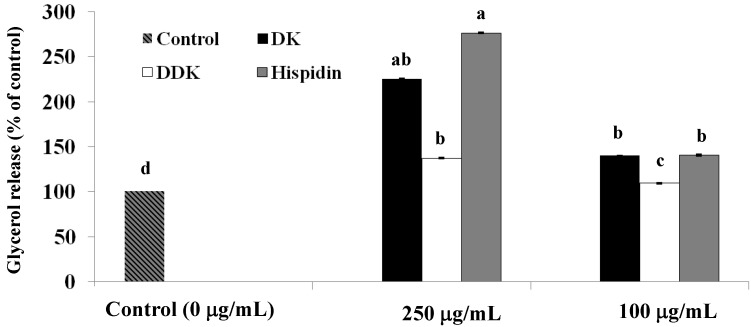
The effect of hispidin, DK and DDK on glycerol release in 3T3-L1 adipocytes. Differentiated 3T3-L1 cells were treated with various concentrations of isolated compounds and incubated for 72 h at 37 °C in a humidified incubator containing 5% CO_2_. Cell supernatants were collected and free glycerol release was assayed. Reported values are the means ± SE (*n* = 3). Letters with different superscripts indicate samples that are significantly different (*p* < 0.05) compared with the control.

To assess whether hispidin, DK and DDK increased lipolysis, the differentiated 3T3-L1 adipocytes were incubated with each compound at concentrations of 100 and 250 µg/mL for 72 h followed by determination of glycerol release. As shown in Figure 4, hispidin, DK and DDK significantly increased glycerol release when compared to the untreated control. At 250 µg/mL, hispidin, DK, and DDK increased glycerol release by 276.4% ± 0.8%, 225.1% ± 0.6%, and 137.3% ± 0.5%, respectively. Even at 100 µg/mL, hispidin, DK, and DDK increased glycerol release by 140.5% ± 0.9%, 139.9% ± 0.4%, and 109.2% ± 0.6%, respectively.

### 2.4. Measurement of Intracellular cAMP

**Figure 5:**
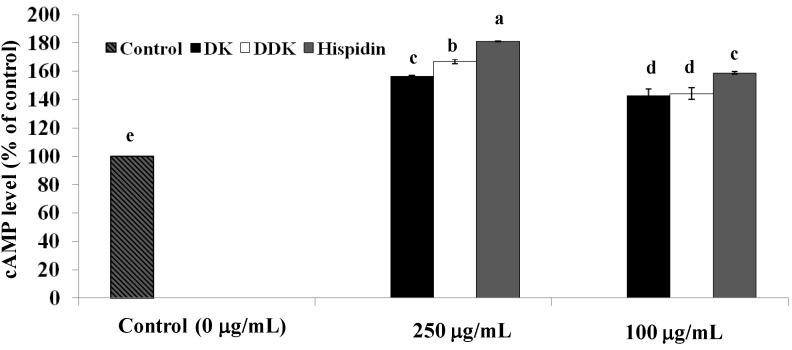
The effect of hispidin, DK and DDK on intracellular cAMP concentrations in 3T3-L1 adipocytes. Differentiated 3T3-L1 cells were treated with various concentrations of isolated compounds and incubated for 72 h at 37 °C in a humidified incubator containing 5% CO_2_. Cell supernatants were collected for cAMP assay. Reported values are the means ± SE (*n* = 3). Letters with different superscripts indicate samples that are significantly different (*p* < 0.05) than the control.

The rate of lipolysis in adipocytes, in general, is critically dependent on the intracellular concentration of cAMP. To examine whether the increased lipolysis induced by hispidin, DK, and DDK involved a change in intracellular cAMP concentration, differentiated 3T3-L1 adipocytes were incubated for 72 h in the absence and presence of the tested compounds, and intracellular cAMP concentrations were determined. We found that intracellular cAMP was significantly increased by all of the compounds (*p* < 0.05) when compared to the untreated sample (Figure 5). At 250 µg/mL of each compound, the intracellular cAMP concentration was increased by 81.2% ± 0.06%, 67.0% ± 1.62%, 56.9% ± 0.19% for hispidin, DDK, and DK, respectively. At 100 µg/mL, cAMP was also significantly increased by 58.8% ± 0.89%, 44.4% ± 4.2%, and 43.1% ± 4.22%, respectively. These results indicated that the increased lipolysis rates caused by hispidin and the compounds from alpinia rhizomes are likely related to a significant increase (*p* < 0.05) in intracellular cAMP concentration.

### 2.5. Evaluation of Lipid Accumulation

**Figure 6:**
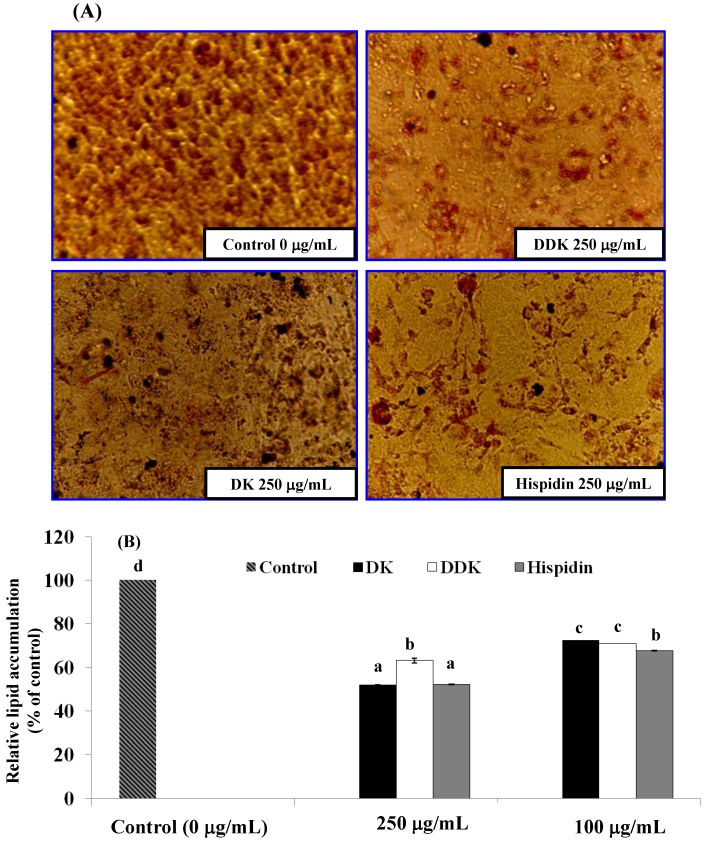
The effect of hispidin, DK and DDK on lipid accumulation in 3T3-L1 adipocytes. (**A**) Effects of hispidin, DK and DDK on lipid droplet formation in 3T3-L1 adipocytes assessed by staining with Oil Red O dye and visualization light microscopy. (**B**) Relative lipid content of each sample determined by quantitative analysis of Oil Red O content. Reported values are the means ± SE (*n* = 3). Letters with different superscripts indicate samples that are significantly different (*p* < 0.05) than the control.

The effect of hispidin, DK and DDK on preventing lipid accumulation in adipocytes was examined by Oil Red O staining. As shown in Figure 6A, hispidin, DK, and DDK inhibited lipid accumulation in a dose-dependent manner. Among the three compounds tested, hispidin and DK reduced lipid droplet size the most when compared with untreated cells. After visualization, lipid accumulation was quantified by dissolution and UV spectroscopy of Oil Red O (Figure 6B). At 250 µg/mL, lipid accumulation was inhibited by 47.8% ± 0.16% and 48.0% ± 0.2% by treatment with hispidin and DK, respectively. This was significantly more inhibition (*p* < 0.05) than was observed for DDK (36.8% ± 1.2%). Thus, hispidin and DK more effectively inhibit lipid accumulation than DDK. Similarly, at 100 µg/mL, hispidin, DK and DDK also significantly inhibited lipid accumulation by 32.3% ± 0.06%, 27.4% ± 0.005%, and 28.0% ± 0.05% compared to the control.

### 2.6. Measurement of Intracellular Triglyceride Content

**Figure 7:**
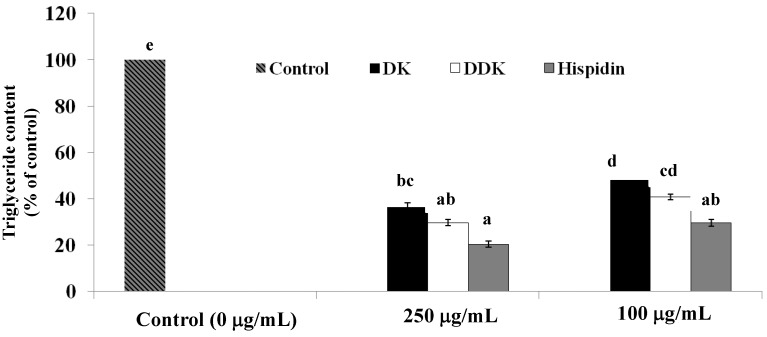
The effect of hispidin, DK and DDK on the inhibition of intracellular triglyceride content in 3T3-L1 adipocytes. Cells were treated with 100 and 250 µg/mL tested compounds for 72 h at 37 °C in a humidified incubator containing 5% CO_2_. Reported values are the mean ± SE (*n* = 3). Letters with different superscripts indicate samples that are significantly different (*p* < 0.05) than the control.

The effect of each compound on intracellular triglyceride content was assessed following treatment for 72 h beginning on day 9 after differentiation. All of the tested compounds significantly reduced intracellular triglycerides in a dose-dependent manner (Figure 7). At 250 µg/mL, hispidin and DDK lowered triglyceride content by 79.5% ± 1.37% and 70.2% ± 1.4%, respectively, (*p* < 0.05) significantly different from the untreated control (*p* < 0.05). To a lesser extent, 250 µg/mL DK also lowered triglyceride content by 63.4% ± 1.7%. At 100 µg/mL, hispidin, DDK and DK also lowered triglyceride content by 70.3% ± 1.54%, 59.1% ± 1.12%, and 51.8% ± 0.05%, respectively.

### 2.7. Glycerol-3-Phosphate Dehydrogenase Inhibition

**Figure 8:**
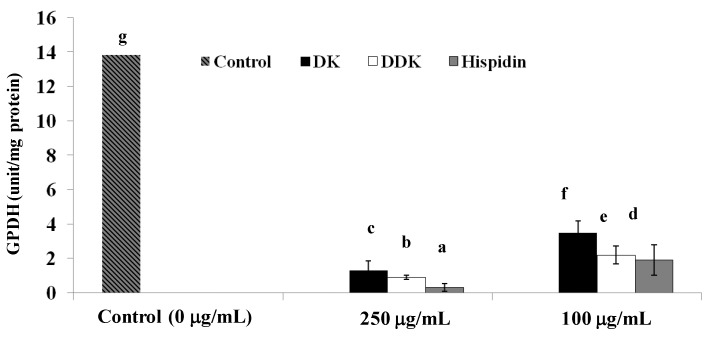
The effect of the hispidin, DK and DDK on the inhibition of GPDH activity in 3T3-L1 adipocytes. Cells were treated with 100 and 250 µg/mL tested compounds for 72 h and incubated at 72 h at 37 °C in a humidified incubator containing 5% CO_2_. Reported values are the mean ± SE (*n* = 3). Letters with different superscripts indicate samples that are significantly different (*p* < 0.05) than the control.

GPDH is an important enzyme for lipid metabolism that is central to triglyceride synthesis. Consequently, one possible mechanism for lowering triglycerides would be to inhibit GPDH. To assess this possibility, the GPDH inhibitory activity of the compounds described herein was assessed. The results showed that 250 µg/mL of hispidin, DDK or DK does significantly inhibit GPDH activity by 97.8%, 94.2%, and 90.5%, respectively. Furthermore, at a lower concentration of 100 µg/mL, hispidin, DDK and DK also inhibited GPDH by 84.7%, 81.1%, and 74.6%, respectively, compared to the control (Figure 8).

### 2.8. Inhibition of Pancreatic Lipase Activity

**Figure 9:**
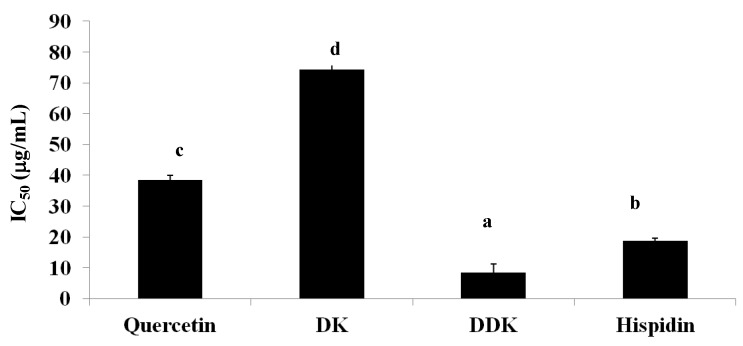
The effect of hispidin, DK and DDK on pancreatic lipase activity. Reported values are the mean ± SE (*n* = 3). Letters with different superscripts indicate samples that are significantly different (*p* < 0.05) than the control.

A recently proposed strategy for reducing the absorption of free fatty acids is to delay triglyceride digestion by inhibiting pancreatic lipase. We found that hispidin, DDK, and DK did have inhibitory activity of pancreatic lipase *in vitro* (Figure 9). DDK was the most potent inhibitor with a 50% inhibitory concentration (IC_50_) of 8.4 ± 2.8 µg/mL, followed by hispidin (IC_50_ = 18.8 ± 0.8 µg/mL) and DK (IC_50_ = 74.4 ± 3.1 µg/mL). For comparison, quercetin, a known lipase inhibitor, had an IC_50_ of 38.5 ± 1.4 µg/mL. Thus, DDK and hispidin appear to be more potent inhibitors of pancreatic lipase *in vitro* than quercetin, suggesting that they could potentially be optimized as pancreatic lipase inhibitors.

### 2.9. Discussion

Obesity increases the risk of multiple medical conditions and is associated with high morbidity and mortality [25]. Adipose tissue mass and adipocyte differentiation have become major research interest for understanding the prevalence and causes of obesity and obesity-related disorders [26]. An article by Wang and Jones suggested that decreasing adipocytic lipogenesis could be a viable strategy for treating and reducing obesity [27].

Natural products have diverse structures and can be important leads for drug discovery [28]. In this study, we investigated the obesity-related *in vitro* activities of hispidin and two related compounds that were isolated from alpinia rhizomes. First, we used an MTT assay to demonstrate that the compounds did not affect 3T3-L1 adipocyte viability. This is consistent with literature data describing the safe use of hispidin as a traditional medicine for multiple maladies [7,9,13,14,29]. Additionally, several potentially beneficial *in vitro* properties have been reported for DK and DDK, with little indication that they are cytotoxic [21,22,23,24].

Lipolysis is of interest to anti-obesity researchers because of its ability to reduce lipid storage. To determine their potential lipolytic effects, glycerol release was measured for hispidin, DK and DDK in 3T3-L1 adipocytes. We found that all three compounds significantly increased lipolysis, indicating that they could have valuable anti-obesity activity. Among the three, hispidin and DK showed the highest levels of glycerol release compared with the control. In addition, we assessed whether the compounds affect the intracellular concentration of cAMP, an important second messenger in fat storage signaling pathways whose concentration is linked to lipolysis [30]. A previous study reported that increasing intracellular cAMP in adipocyte cells can stimulate lipolysis [31]. The results from our study revealed that hispidin, DK and DDK all increase intracellular cAMP in 3T3-L1 adipocytes, perhaps explaining why they stimulate lipolysis. These data also support the potential for developing the compounds as anti-obesity therapeutics.

Next, we examined the effects of the compounds on the inhibition of lipid accumulation. Hispidin and DK strongly decreased lipid droplets size in 3T3-L1 adipocytes compared to DDK and the untreated control. This trend was confirmed by quantitative spectrophotometric analysis of neutral lipid content. Thus, hispidin and DK inhibit lipid accumulation in 3T3-L1 adipocytes.

The prevention of triglyceride absorbtion has also been explored as a therapeutic strategy for obesity treatment [32]. To further evaluate the therapeutic potential of the tested compounds, their effect on intracellular triglyceride content was determined. The results indicated that hispidin, DK and DDK significantly reduced triglyceride content in 3T3-L1 adipocytes in a dose-dependent manner. Moreover, all three compounds inhibit the activity of GPDH for a key enzyme in the conversion of glycerol to triglycerides [33]. Hispidin and DDK were the most potent GPDH inhibitors, with DK also showing significant inhibition. The important role of GPDH in converting preadipocytes to adipocytes [34] further underscore to the importance of this activity and the potential of the compounds.

The inhibition of dietary fat absorption is also a logical target for managing obesity, and pancreatic lipase is a key enzyme involved in triglyceride absorption in the small intestine [35]. Since inhibitors of digestive lipase should be useful anti-obesity agents, recent research has focused on identifying novel and safe lipase inhibitors from natural sources like phytic acid, tannin, saponins and Oolong tea [36,37,38,39]. The results from our study indicated that hispidin, DK and DDK do inhibit pancreatic lipase, consistent with the activity reported by other groups [40,41]. Thus, in addition to their other favorable activities, hispidin, DK and DDK could potentially prevent obesity by inhibiting pancreatic lipase.

## 3. Experimental Section

### 3.1. General

*p*-Nitrophenyl butyrate (NPB), lipase (type II: from porcine pancreas), MTT (3-(4,5-dimetyl-2-thiazolyl)-2,5-diphenyltetrazoliumbromide), insulin, the free glycerol determination kit, the glycerol standard, morpholinepropanesulphonic acid (MOPS), Oil Red O, cAMP enyme immunoassay kit, and hispidin were purchased from Sigma Aldrich Chemical Co. (St. Louis, MO, USA). 3T3-L1 cells were obtained from the American Type Culture Collection (ATCC, Rockville, MD, USA). Dulbecco’s Modified Eagle Medium (D-MEM), calf serum (CS), fetal bovine serum (FBS), ethylenediaminetetraacetic acid (EDTA), dexamethasone, 3-isobutyl-1-methyl xanthine (IBMX), and calcium chloride were purchased from Wako Pure Chemical Industries, Ltd. (1-2, Doshomachi 3-Chome, Osaka, Japan). 2-Amino-2-hydroxymethyl-propane-1,3-diol (Tris) was obtained from Kanto Chemical. Co. Inc. (2-8, Nihonbashi Honcho-3 chome, Tokyo, Japan). The triglyceride colorimetric assay kit was purchased from Cayman Chemical. Co. (Ann Arbor, MI, USA). Glycerol-3-phosphate dehydrogenase (GPDH) was obtained from BioVision, Inc. (Milpitas, CA, USA). All reagents were of the highest grade available.

### 3.2. Preparation of Rhizomes Extracts and Isolation of DK and DDK

The isolation of DK and DDK were isolated from rhizomes of alpinia as reported previously [22]. The rhizomes of alpinia were collected from the campus of the University of the Ryukyus in Okinawa. To isolate the DK and DDK, 500 g of fresh alpinia rhizome was boiled in 3.5 L of water for 20 min. After cooling, the mixture was filtered, and the filtrate was reduced to 1 L under vacuum at 40 °C and extracted three times with 500 mL of hexane. The combined hexane fraction was evaporated to dryness under vacuum. The dried residue was then boiled in water, and the mixture was filtered while hot. To isolate DK, the retentate was purified by preparative high performance liquid chromatography (HPLC) using a TSK gel ODS-100Z column (15 × 0.46 cm i.d., 5 µm particle size; Tosoh Corp., Tokyo, Japan). The mobile phase was 0.1% aqueous acetic acid at a flow rate of 0.8 mL/min with a gradient of increasing methanol (MeOH) from 50% to 100% over 20 min. To isolate DDK, the hot filtrate was cooled to 4 °C to allow recrystallization, and the resulting crystals were further purified using preparative HPLC with the same method as for DK purification. The quantification and purification of DK and DDK by HPLC are shown in Figure 2.

### 3.3. Cell Culture and Differentiation

3T3-L1 cells were grown to confluency in Dulbecco’s modified eagle’s medium (DMEM) with 2% glutamine and 10% CS (v/v). Two days after reaching confluency, the cells were stimulated to differentiate into adipocytes by growing for an additional two days in DMEM that contained 10% FBS, 0.5 mM IBMX, 1 µM dexamethasone, and 10 µg/mL insulin. Cells were then maintained in DMEM with 10% FBS and 10 µg/mL insulin for another two days, followed by culturing in DMEM with only 10% FBS for an additional four days. At that time, greater than 90% of the cells were differentiated 3T3-L1 adipocytes with accumulated lipid droplets. Differentiated 3T3-L1 cells were treated with different concentrations of the test compounds and maintained at 37 °C in a humidified incubator containing an atmosphere of 5% CO_2_ throughout the experiments.

### 3.4. Cell Viability Assays

The effects of hispidin and the compounds from alpinia rhizomes on cell viability were determined by MTT assay [42]. Differentiated 3T3-L1 adipocytes were incubated for 72 h in the presence of test compounds at concentrations of 100 and 250 µg/mL 80 µL of a 5 mg/mL solution of MTT in phosphate buffered saline (PBS) was added to each well, followed by incubation at 37 °C for 4 h. After incubation, the medium was removed and the resulting formazan crystals were dissolved in 200 µL of 0.04 M HCl in isopropanol. After incubating at 37 °C for 30 min with gentle shaking, the mixture was centrifuged at 13,000 *g* for 2 min and the supernatants were aspirated. Absorbance was measured at 570 nm using a multi-well plate reader. For each treatment, cell viability was calculated as a percentage (%) using the following formula:

(%) of cell viability = (Abs. of treated sample/Abs. of untreated samples) × 100
(1)

### 3.5. Determination of Glycerol Release

Glycerol release was examined according to a previously described method [43]. Differentiated 3T3-L1 cells were incubated for 72 h in the presence of test compounds at concentrations of 100 and 250 µg/mL. Glycerol in the medium was measured using a free glycerol determination kit with glycerol standards used for calibration. Briefly, 200 µL of the free glycerol reagent reconstituted in distilled water was mixed with 50 µL of distilled water (blank), glycerol standard, or a test samples that included cells. Mixtures were then incubated at 37 °C for 15 min, and the absorbance of the solution was measured at 540 nm using a microplate reader. The glycerol content was calculated by the formula:

glycerol content = ([Abs. of sample − Abs. of blank]/[Abs. of standard − Abs. of blank]) × concentration of standard
(2)

### 3.6. Measurement of Intracellular cAMP

The cAMP concentration was measured using a cAMP immunoassay kit [44]. Briefly, differentiated 3T3-L1 adipocytes were lysed in 0.1 M HCl to inhibit phosphodiesterase activity. The supernatants were then collected, neutralized, and diluted, after which a fixed amount of cAMP conjugate was added to the mixtures to compete with cell lysate-derived cAMP for binding to rabbit polyclonal antibodies immobilized on a 96 well plate. After washing the plates to remove excess conjugated and unbound cAMP, a substrate solution was added to the wells to determine the activity of the bound enzyme. The color development was the topped, after which the absorbance was read at 415 nm. The intensity of the absorbance was inversely proportional to the concentration of cAMP in the cell lysate.

### 3.7. Evaluation of Lipid Accumulation by Oil Red O Staining

Intracellular lipid accumulation was measured using Oil Red O [45]. Differentiated 3T3-L1 cells were incubated for 72 h in the presence of test compounds at concentrations of 100 and 250 µg/mL. Cells were washed twice with PBS and then fixed with formalin for 1 h at room temperature. Fixed cells were washed twice with water and once with 60% isopropanol in water, followed by incubation with Oil Red O working solution for 3 h. The stained 3T3-L1 adipocyte preparations were rinsed four times with distilled water. Representative images of treated cells were obtained with an Olympus microscope (Yashima Optical Co.; LTD, Tokyo, Japan). For quantification, stained cells were dissolved in isopropanol, and the absorbance at 500 nm was measured with a multi-well plate reader. The Oil Red O intensity in treated samples (hispidin, DK, DDK) was calculated relative to untreated samples using the following equation:

% intensity = (Abs_treatment_/Abs_control_) × 100 (3)

where Abs_control_ is the absorbance of the untreated sample and Abs_treatment_ is the absorbance of the treated sample.

### 3.8. Measurement of Triglyceride Content

3T3-L1 adipocytes were harvested 9 days after the initiation of differentiation. Cells were incubated in the presence of test compounds at concentrations of 100 and 250 µg/mL for 72 h at 37 °C in a humidified incubator with an atmosphere of 5% CO_2_. Treated cells were collected and lysed in lysis buffer (1%Triton X-100 in PBS), and the total triglyceride content in cells was determined using a commercial triglyceride assay kit (DiaSys Diagnostic Systems GmbH, Holzheim, Germany).

### 3.9. Glycerol-3-Phosphate Dehydrogenase Assay

Glycerol-3-phosphate dehydrogenase (GPDH) activity was determined according to the procedure of Wise and Green [33]. 3T3-L1 adipocytes were harvested 9 days after the initiation of differentiation. Cells were incubated in the presence of test compounds at concentrations of 100 and 250 µg/mL for 72 h at 37 °C in a humidified incubator with an atmosphere of 5% CO_2_. Treated cells were carefully washed twice with ice-cold PBS and lysed in 25 mM Tris/1 mM EDTA pH 7.5 for the measurement of glycerol-3-phosphate dehydrogenase (GPDH) specific activity, which was determined by measuring the amount of NADH product at 450 nm using microplate reader and incubated for 72 h. The protein concentration was determines by the BioRad DC protein assay kit (Bio-Rad Laboratories, Hercules, CA, USA) using bovine serum albumin as a standard.

### 3.10. Pancreatic Lipase Assay

Pancreatic lipase activity was measured according to the procedure reported by Kim *et al.* [46] with slight modifications. Briefly, an enzyme buffer was prepared by the addition of 10 µL of a 2.5 mg/mL solution of porcine pancreatic lipase in 10 mM MOPS and 1 mM ethylenediaminetetraacetic acid (EDTA), pH 6.8 to 170 µL of Tris buffer (100 mM Tris-HCl and 5 mM CaCl_2_, pH 7.0). Each tested compound (20 µL) at concentrations of 10, 100, and 250 µg/mL was mixed with 20 µL of the enzyme buffer, followed by incubation for 15 min at 37 °C. 5 µL of the 10 mM p-nitrophenyl butyrate (p-NPB) substrate in dimethyl formamide was added, and the reactions were incubated for 30 min at 37 °C. Lipase activity was determined by monitoring the hydrolysis of p-NPB to p-nitrophenol via UV detection at 405 nm using a microplate reader. Inhibition of lipase activity (% Inhibition) was expressed as a percentage decrease in optical density for samples that contained test compound compared to untreated samples. % Inhibition was calculated as follows:

% Inhibition = 100 − (B − b/A − a) × 100
(4)

where A is the activity without inhibitor, a is the activity of the negative control without inhibitor, B is the activity with inhibitor, and b is the activity of the negative control with inhibitor.

### 3.11. Statistical Treatment

All assays were performed in triplicate. The data for cell viability, glycerol release, intracellular cAMP, triglyceride content, GPDH and pancreatic lipase were evaluated using an analysis of variance (ANOVA) test followed by Duncan’s test. The data are presented as the mean ± standard error. A *p* value < 0.05 was considered statistically significant.

## 4. Conclusions

In conclusion, our study indicated that hispidin and two additional compounds isolated from alpinia rhizomes exhibited several pharmacological activities that could be beneficial as anti-obesity treatments. Specifically, these compounds increased intracellular cAMP and glycerol release, inhibited lipid accumulation, and reduced triglyceride content. Moreover, they inhibit GPDH and pancreatic lipase and have no measurable cytotoxic activity toward 3T3-L1 adipocytes *in vitro*. In an aggregate, our results demonstrate that hispidin, DK and DDK have the strong potential as anti-obesity optimization leads.
